# Can quantum-chemical NMR chemical shifts be used as criterion for force-field development

**DOI:** 10.1186/1758-2946-6-S1-O2

**Published:** 2014-03-11

**Authors:** Thomas E Exner, Andrea Frank, Heiko M Möller, Martin Dračínský

**Affiliations:** 1 Institute of Pharmacy, University of Tübingen, 72076 Tübingen, Germany; 2 Department of Chemistry, University of Konstanz, 78457 Konstanz, Germany; 3 Institute of Organic Chemistry and Biochemistry, Academy of Sciences, 166 10 Prague, Czech Republic

## 

Fragment-based quantum chemical calculations based on our adjustable density matrix assembler (ADMA) are able to calculate NMR chemical shifts even for proteins and protein-protein complexes [1,2]. The agreement between the calculated and experimental chemical shifts in these calculations is, however, highly dependent on including conformational sampling and explicit solvent molecules. On the one hand, ensembles taken from classical MD simulations are suited for ^13^C and ^1^H chemical shift calculations if polar protons are neglected [3]. On the other hand, input structures from a Car–Parrinello MD resulted in landmark improvements over calculations based on classical MD especially for amide protons, which are predicted too high-field shifted based on the latter ensembles [4]. The better results are caused by the solute–solvents interactions forming shorter hydrogen bonds as well as by the internal degrees of freedom of the solute. With the obtained accuracy and the possibility of identifying the structural reasons for discrepancies between the experimental and calculated data, NMR chemical-shift calculations are now a perfect tool for e.g. the validation of new, improved force fields.
